# Osteoid Cancer of the Femur

**Published:** 1860-10

**Authors:** E. Andrews


### OSTEOID CANCER OF THE FEMUR.

The tumor in question, was forwarded to Prof. N. S. Davis, of Lind University, by Drs. L. Martin and E. C. Dickenson, of Shelbyville, Ill.

The history of the patient is given in the following letter from Dr. Dickenson :

"Patient, William, aged 10 years, son of Jesse Baker, a farmer residing in the South Eastern part of Shelby Co., near Neoga, Ill. About the middle of June the father called upon us for advice in the case, as stated by him. We requested an examination, for which an opportunity was afforded by his presentment at our office on Saturday, June 30th. On that day we diagnosed a tumor occupying the right knee, and having a circumference of 2 feet 2½ inches. The patient, of an ambitious sanguine temperament, and a champion among his school fellows, in their sports, had acquired the reputation of the best hopper opon one foot in the settlement, and it was in February, 1859, without having experienced previous known injury, that the inner condyle of the right femur began to be tender to the touch. Soon, also, it appeared swollen, and continued gradually to increase in size, though without offering serious impediment to locomotion.

The patient continued his wonted state of health until March or April last, when, as the tumor took on an increased activity of growth, he began to lose flesh, and during the three weeks previous to our seeing him, (June 30th,) had rapidly emaciated. The pain had become constant and of increased intensity. Comparing then the alternatives of probable dissolution from exhaustion, within three or four weeks, with the improbable immediate death, as the result of a carefully conducted amputation, we determined to operate on the next day but one, (July 20th.) Accordingly we took the cars for Neoga, invited the assistance of two physicians of that place, and proceeded at once five miles, and to the operation. I prepared Prof. Andrews' anaesthetic compound, *i. e.* Ether 2 pts., Chlor. 1 pt., and occupied ten minutes in preparing the patient for the knife in the hands of Dr. Martin. Completed the operation with pulse at 95. One hour after the operation, pulse at 120. Employed two ¼ gr. doses Morph. which controlled all pain and procured sleep within two hours. 'Six hours afterward, slept quietly—pulse 130.'

I remained with him for two days, during which time he constantly improved in strength and spirits. Visited him again in four days, and dressed the stump. In five days more Dr. M. visited and removed sutures. Left ligatures for removal by the patient after 14 or 15 days. Saw the patient no more, but heard from his father that in 14 days from operation he was moving about on crutches, and in 21 days was visiting the neighbors on horseback.

In five weeks after the operation he commenced attending school 1½ miles, and has attended *constantly* since."

When the specimen came into my hands for examination, it was too far gone in decomposition to permit any cancer elements to be detected by the microscope, and the examination therefore was made by simple dissection. The specimen consisted of a thigh and leg. At the place of the knee joint there was a large tumor measuring eight inches in one diameter and fourteen in the other. The muscles above and below were very much atrophied. The integument over nearly the whole tumor was natural in appearance, and not strongly adherent. On removing the skin and superficial fascia, I came at once to a mass which seemed like an encephaloid cancer on the surface, but which, at the depth of a quarter of an inch rested on a loose bony tissue mixed with cancerous substances. As the dissection was pushed deeper, the bony tissue seemed firmer and more compact, and less mixed with soft tissue. The mass sprang exclusively from the femur. The patella, and the upper part of the tibia, though over-lapped and pressed upon by the tumor, yet retained their coating of cartilage, and a nearly natural appearance. The articular surface of the femur also retained a pretty natural aspect, but the whole surface of the bone just above was over-lapped in a semi-fibrous mass, fibres of osseous tissue seeming to spring from the whole circumference and radiate in all directions, enveloping the shaft of the bone some two inches above the point of attachment, giving the appearance of the shaft of the bone running into a hole or socket in the tumor.

The case so far as could be determined by this examination, was one of osteoid cancer.

E. ANDREWS.

